# Caracterización genómica de los mecanismos de adaptación de *Pseudomonas aeruginosa* en pacientes con bronquiectasias

**DOI:** 10.1515/almed-2025-0173

**Published:** 2025-12-08

**Authors:** Alba Muñoz Santa, Xavier Gómez-Arbonés, Ricardo Pifarré Teixidó, Mercè García-González, Alba Bellés Bellés

**Affiliations:** Servicio de Análisis Clínicos, 16296 Hospital Universitari Arnau de Vilanova de Lleida, Institut de Recerca Biomèdica de Lleida (IRB Lleida), Universitat de Lleida, Lleida, Catalunya, España; Departamento de Medicina y Cirugía, Institut de Recerca Biomèdica de Lleida (IRB Lleida), Universitat de Lleida, Lleida, Catalunya, España; Servicio de Neumología, Hospital Universitari Arnau de Vilanova de Lleida, Lleida, Catalunya, España

**Keywords:** bronquiectasias, *Pseudomonas aeruginosa*, adaptación genómica

## Abstract

**Objetivos:**

*Pseudomonas aeruginosa* es una de las principales causas de infección bronquial crónica (IBC), especialmente en pacientes con enfermedades como fibrosis quística (FQ), enfermedad pulmonar obstructiva crónica (EPOC), asma y bronquiectasias (BQ). Si bien la IBC en el contexto clínico de la FQ ha sido objeto de numerosos estudios, la literatura existente en el caso de IBC y BQ es mucho más escasa. El objetivo del presente estudio es determinar el perfil de sensibilidad antibiótica de 100 aislados bacterianos identificados en 100 pacientes con bronquiectasias e infección bronquial crónica por *Pseudomonas aeruginosa*, así como caracterizar algunos de los mecanismos de adaptación en 55 aislados, mediante secuenciación del genoma completo (WGS, por sus siglas en inglés).

**Métodos:**

La sensibilidad antibiótica frente a 10 antipseudomónicos se determinó utilizando el sistema de microdilución en caldo MicroScan WalkAway. La WGS se llevó a cabo con el kit de preparación de librerías de Illumina. La secuenciación de las librerías indexadas se realizó en un secuenciador MiSeq benchtop de Illumina, generando lecturas pareadas de 300 pares de bases.

**Resultados:**

La mayor parte de las mutaciones de pérdida de función se encontraron c en los genes que codifican el sistema de la bomba de expulsión MexAB-OprM, el clúster génico pvd y el receptor FpvA, así como en los genes involucrados en la motilidad tipo *twitching*, como *chpA* y *fimV*.

**Conclusiones:**

Los datos obtenidos indican que *Pseudomonas aeruginosa* se adapta a la IBC mediante la acumulación de mutaciones de pérdida de función en diversos genes que que derivan en diferentes fenotipos y podrían orientar el desarrollo de nuevas terapias alternativas.

## Introducción

*Pseudomonas aeruginosa* es un patógeno oportunista responsable de las causas más frecuentes de infección nosocomial y afecta en mayor medida a los pacientes inmunodeprimidos. Asimismo, *P*. *aeruginosa* es uno de los principales agentes causantes de la infección bronquial crónica (IBC)que contribuye a la morbimortalidad de los pacientes con enfermedades inflamatorias crónicas, como la fibrosis quística (FQ), la enfermedad pulmonar obstructiva crónica (EPOC) o las bronquiectasias (BQ) [1].

Las BQ se caracterizan por la dilatación permanente y destrucción gradual de las paredes bronquiales, como resultado de diversas patologías sistémicas o localizadas [2].

Los estudios que abordan la caracterización genómica de *P*. *aeruginosa* en pacientes con BQ son muy escasos [3] y la mayoría de los publicados hasta la fecha se centran en los mecanismos de resistencia a los antibióticos [4].

El objetivo del presente estudio es determinar la sensibilidad antibiótica de 100 aislados de *P*. *aeruginosa* obtenidos de 100 pacientes con BQ no debida a FQ , así como caracterizar otros mecanismos genéticos de tipo adaptativo diferentes a los de la resistencia antibiótica, que contribuyen a la persistencia de este patógeno en la IBC.

## Materiales y métodos

### Colección de *P*. *aeruginosa* y análisis de sensibilidad antibiótica

La colección estudiada reune 100 aislados de *P*. *aeruginosa* procedentes de muestras respiratorias de 100 pacientes diferentes con BQ no debidas a FQ, atendidos de forma consecutiva en el Hospital Universitari Arnau de Vilanova de Lleida (Cataluña, España) entre septiembre de 2019 y mayo de 2021.

Los pacientes incluidos tenían diagnóstico de bronquiectasias mediante tomografía computarizada de alta resolución (HRCT) y síntomas clínicos compatibles. En dicha cohorte, las etiologías más frecuentes de BQ fueron EPOC (52 %), causas idiopáticas (26 %), otras patologías pulmonares (18 %), y enfermedades genéticas como discinesia ciliar primaria (DCP) (3 %) y síndrome de Kartagener (1 %).

La concentración mínima inhibitoria (CMI) de piperacilina/tazobactam, ceftazidima, cefepima, imipenem, meropenem, tobramicina, amikacina, ciprofloxacino y colistina se obtuvo mediante microdilución en caldo en el sistema MicroScan WalkAway^®^ (Siemens, Healthcare). Las CMI de los aislados de *P. aeruginosa* con fenotipo mucoide y/o que mostraran un perfil de hipersensibilidad antibióticase determinaron mediante tiras Etest^®^(BioMérieux). En relación a la interpretación de las categorías S/I/R, se aplicaron los valores clínicos establecidos en EUCAST v14.0. Los aislados multirresistentes se identificaron siguiendo las recomendaciones de la ECDC [5].

### Caracterización genómica de los aislados de *P. aeruginosa*

Con objetivo de representar la variabilidad genética de *P*. *aeruginosa* en bronquiectasias, se secuenciaron55 aislados con diferetes morfotipos [mucoides, no mucoides, y variantes de aislado pequeña (VCP)] y con perfiles diferentes de sensibilidad antibiótica, dado que la mayor parte de los aislados recogidos fueron sensibles a la mayoría de los antimicrobianos testados. Así mismo, se excluyeron los aislados productoras de carbapenemasas o piomelanina, dado que se trata de subpoblaciones probablemente ya adaptadas.

La extracción del ADN genómico total se llevó a cabo utilizando un kit comercial (QIAsymphony DSP DNA Kit, QIAGEN), en el extractor automático QIAsymphony (QIAGEN). La secuenciación genómica se llevó a cabo mediante la preparación de librerías indexadas de extremos *paired-end* (Illumina DNA Prep, Illumina) con la posterior secuenciación en el sistemaMiSeq^®^ de Illumina, utilizando el kit de reactivos MiSeq v3 y 600 ciclos. Para la caracterización genómica, las lecturas *paired-end* de 300 pb se ensamblaron *de novo *mediante el ensamblador SPAdes, uilizando los parámetros predeterminados, con el fin de inferir el secuenciotipo (ST) mediante la herramienta MLST v2.0.4.(disponible en http://www.genomicepidemiology.org/services). El análisis de detección de variantes (en inglés, *variant calling* -VC) se realizó con el programa Snippy v.3.1 [6] utilizando el genoma PAO1 (NC_002516.2) como referencia. Se aplicaronlos parámetros predeterminados, incluyendo una calidad mínima de mapeo de 60, un umbral de calidad de base de 13, y una cobertura mínima de 10 lecturas, considerando que la variante es válida si hay concordancia en el 90% de las lecturas. La detección de grandes delecionesse realizó con la herramienta Integrative Genomics Viewer (IGV).

## Resultados

**Figura 1:**
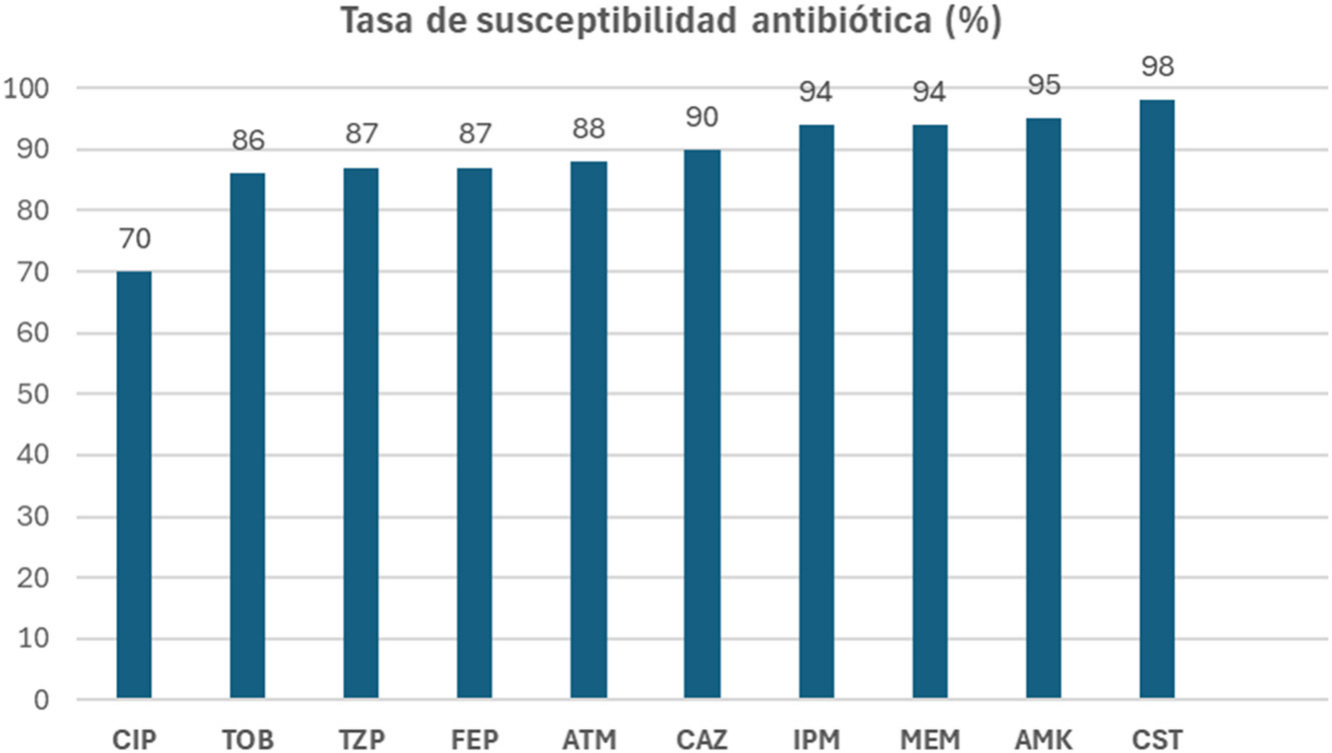
Sensibilidad antibiótica (%) en la colección de *P. aeruginosa*-BQ. CIP, ciprofloxacino; TOB, tobramicina; TZP, piperacilina/tazobactam; FEP, cefepime; ATM, aztreonam; CAZ, ceftazidima; IPM, imipenem; MEM, meropenem; AMK, amikacina; CST, colistina.

En la Figura 1 se muestran los porcentajes de sensibilidad antimicrobiana de los 100 aislados de *P. aeruginosa.*

El antimicrobiano con menor tasa de sensibilidad fue ciprofloxacino, mientras que la colistina, la amikacina y los carbapenémicos fueron los compuestos con mayor actividad. La mayoría de los aislados secuenciados se identificaron como hipersensibles a casi la totalidad de los antibióticos testados, salvo siete aislados que se identificaron como multirresistentes (MDR). Estos aislados MDR se indican en la Tabla 1, donde se detalla el antibiograma de todos los aislados secuenciados.

**Tabla 1:** Perfil de sensibilidad antibiótica de los 55 aislados secuenciados.

CMI, mg/L^a^											
Aislado	ST	ATM	TZP^b^	CAZ	FEP	IMI	MER	TOB	AK^b^	CIP	CST
BQ01	1251	0*,*75	≤8	1*,*5	4	0*,*75	0*,*125	2	≤8	1*,*5	1
BQ02	244	0*,*016	≤8	0*,*047	4	0*,*75	0*,*5	0*,*032	≤8	>32	1
BQ03	155	0*,*125	≤8	0*,*38	3	0*,*50	3	0*,*75	≤8	0*,*016	1*,*5
BQ04	242	1*,*5	≤8	1*,*5	4	2	0*,*38	2	16	1*,*5	1
BQ05	262	3	≤8	1*,*5	4	0*,*75	0*,*25	1	≤8	1*,*5	1
BQ06	395	3	≤8	3	0,75	6	2	0*,*28	≤8	0*,*38	3
BQ07^c^	348	12	≤8	24	0,75	1*,*5	0*,*75	8	32	1	1
BQ08	598	0*,*25	≤8	1	1,5	0*,*75	0*,*38	0*,*75	≤8	0*,*38	1
BQ09	170	0*,*19	≤8	0*,*5	0,75	1*,*5	0*,*125	1*,*5	≤8	0*,*064	2
BQ10	3016	1*,*5	≤8	1	4	0*,*75	0*,*25	1	≤8	0*,*5	1*,*5
BQ11^c^	UK	0*,*50	24	2	16	0*,*75	0*,*064	2	≤8	3	1
BQ12	395	0*,*25	≤8	1	8	1	0*,*064	1*,*5	16	0*,*38	1
BQ13	253	0*,*25	≤8	0*,*50	≤1	1*,*5	0*,*125	1*,*5	≤8	>32	1
BQ14	633	1*,*5	≤8	1	4	2	0*,*50	2	≤8	2	2
BQ15	2431	2	64	1	1	2	0*,*125	1	≤8	0*,*094	2
BQ16^c^	175	12	64	16	12	>32	>32	>256	≤8	>32	2
BQ17	532	3	≤8	0*,*75	1	1*,*50	1	0*,*75	≤8	1	1
BQ18	554	0*,*25	≤8	0*,*50	4	4	1	0*,*75	≤8	0*,*50	2
BQ19	253	1	≤8	0*,*50	0*,*75	1	0*,*38	1	≤8	0*,*064	1
BQ20	UK	0*,*094	≤8	0*,*125	0*,*047	0*,*064	0*,*008	0*,*50	≤8	0*,*008	1
BQ21	480	0*,*38	≤8	0*,*38	0*,*5	0*,*38	0*,*064	0*,*064	≤8	0*,*19	1*,*5
BQ22	UK	0*,*38	≤8	1	1*,*5	3	0*,*032	1*,*5	≤8	0*,*064	1
BQ23	357	4	≤8	1	2	1	0*,*125	2	≤8	0*,*125	3
BQ24	2431	0*,*5	≤8	0*,*5	1	0*,*75	0*,*064	1*,*5	≤8	0*,*094	1*,*5
BQ25	1600	2	≤8	1	1	3	0*,*75	1	≤8	0*,*125	1
BQ26^c^	UK	1*,*5	64	6	2	>32	>32	0*,*50	≤8	>32	1
BQ27	379	2	≤8	0*,*75	0*,*75	1*,*5	0*,*19	1	≤8	0*,*094	2
BQ28^c^	633	>256	≤8	8	>256	1*,*5	0*,*75	>256	≤8	>32	2
BQ29	274	0*,*047	≤8	0*,*094	0*,*25	0*,*25	0*,*012	0*,*047	≤8	2	0*,*25
BQ30	253	0*,*25	≤8	0*,*38	0*,*75	0*,*38	0*,*047	0*,*75	≤8	0*,*023	1
BQ31	1637	3	≤8	1	1	1	0*,*25	1	≤8	0*,*094	1*,*5
BQ32	253	4	≤8	1	1	1	0*,*50	1	≤8	0*,*094	4
BQ33	360	0*,*50	≤8	2	12	1	0*,*049	3	32	0*,*25	1
BQ34	1002	0*,*38	≤8	0*,*75	0*,*20	1	0*,*094	0*,*75	≤8	0*,*035	2
BQ35	3119	3	≤8	1*,*5	16	0*,*19	0*,*094	2	32	0*,*25	1
BQ36	262	4	≤8	3	3	3	0*,*50	2	≤8	0*,*125	3
BQ37	447	2	≤8	1	1*,*5	2	0*,*50	1	≤8	0*,*094	1*,*5
BQ38	UK	3	≤8	1	1	1*,*5	0*,*125	1	≤8	0*,*023	1
BQ39	585	0*,*19	≤8	0*,*75	0*,*25	1*,*5	0*,*023	0*,*25	≤8	0*,*023	1
BQ40	3218	1*,*5	≤8	0*,*75	1	0*,*75	0*,*094	0*,*75	≤8	0*,*064	1
BQ41	598	0*,*19	≤8	0*,*5	6	0*,*38	0*,*19	0*,*25	≤8	0*,*38	1
BQ42	395	0*,*094	≤8	0*,*125	0*,*094	0*,*094	0*,*012	0*,*064	≤8	0*,*008	1
BQ43	17	0*,*25	≤8	0*,*094	0*,*125	0*,*125	0*,*012	0*,*094	≤8	0*,*047	0*,*064
BQ44	UK	2	≤8	0*,*75	0*,*75	0*,*75	0*,*064	0*,*125	≤8	0*,*047	0*,*064
BQ45	253	0*,*380	≤8	0*,*75	8	0*,*250	0*,*064	3	32	0*,*5	2
BQ46	792	0*,*75	≤8	0*,*38	2	1	0*,*094	0*,*75	≤8	0*,*38	1
BQ47	3511	16	≤8	3	4	2	8	0*,*125	≤8	2	2
BQ48	1393	4	≤8	2	4	2	0*,*38	1	≤8	0*,*125	1*,*5
BQ49	455	0*,*38	≤8	1	0*,*25	2	0*,*094	1	≤8	0*,*064	1
BQ50	253	2	≤8	1	8	1*,*5	0*,*5	1	≤8	>32	1*,*5
BQ51	253	4	≤8	1	0*,*094	1	0*,*75	1*,*5	≤8	>32	1
BQ52	27	0*,*125	≤8	0*,*25	4	0*,*125	0*,*094	0*,*5	32	>32	1
BQ53^c^	UK	64	>256	96	24	2	0*,*75	1	≤8	0*,*064	1
BQ54	274	3	≤8	4	4	1*,*5	0*,*75	1	≤8	0*,*50	1
BQ55^c^	235	48	64	32	24	1	0*,*75	2	≤8	>32	1

Concentración mínima inhibitoria (CMI) (mg/L)^a^ de ATM, aztreonam (S≤0*,*001; R>16); TZP, piperacilina/tazobactam (S≤0*,*001; R>16); CAZ, ceftazidima (S≤0*,*001; R>8); FEP, cefepime (S≤0*,*001; R>8); IMI, imipenem (S≤0*,*001; R>4); MER, meropenem (S≤2; R>8); TOB, tobramicina (S≤2; R>2); AK, amikacina (S≤16; R>16); CIP, ciprofloxacino (S≤0*,*001; R>0,5); CST, colistina (S≤4; R>4). Las CMI se determinaron mediante E-test, a excepción de los marcados con^b^, que se determinaron mediante microdilución en caldo. El color verde indicasensibilidad clínica a dosis estándarl y a dosis incrementada, mientras que el color rojo indica resistencia. ^c^Indica que se trata de un aislado MDR; la columna ST represeta el secuenciotipo; UK es indicativo de ST no conocido.

Para la caracterización de los mecanismos de adaptabilidad, se realizó el análisis de VC en los 55 aislados secuenciados, con el objeto de identificar mutaciones de pérdida de función *(loss-of-function mutations).*

En la Tabla 2 se muestran los ST y las mutaciones de pérdida de función identificadas en los diferentes genes asociados a la adaptabilidad de *P*.* aeruginosa* al ambiente pulmonar crónico.

**Tabla 2:** Secuenciotipo (ST) y mutaciones de pérdida de función identificadas en la bomba de expulsión MexAB-OprM, *mutS*-*mutL, mucA-mucB;* clúster psl, genes *bifA-rbdA-oprF-ladS* y *pilJ-chpA-fimV* de cada uno de los aislados secuenciados.

Aislado	ST	MexAB-OprM	*mutS-mutL*	*mucA-mucB*	psl cluster	*bifA-rbdA-oprF-ladS*	*pilJ-chpA-fimV*
BQ01^b^	1251	*mexA* (nt30Δ2)	–	*mucA* (Q123X)	–	–	*chpA* ^c^ *, fimV* ^c^
BQ02^b^	244	*oprM* (Q246X)	–	*mucA* (Q118X)	ΔpslA-pslO (≈104 kb)	–	–
BQ03^b^	155	–	–	*mucA* (V147X)	ΔpslA-pslO (≈135 kb)	–	–
BQ04^a^	242	–	–	*mucA* (A90X)	–	–	–
BQ05^a^	262	–	–	*mucA* (Q117X)	ΔpslE-pslN (≈132 kb)	*oprF* (nt576Δ5, nt844Δ1)	–
BQ06^a, b^	395	*oprM* (nt800Δ1)	–	*mucA* (H159X)	ΔpslA-pslO (≈142 kb)	–	–
BQ07^a^	348	*oprM* (aas202InsHis)	–	*mucA* (V99X)	ΔpslB-pslC (≈1.8 kb)	*ladS* (aas497Δ6)	–
BQ08^a^	598	*mexA* (Q107X)	–	*mucA* (V147X)	–	–	–
BQ09	170	*mexA* (Q295X)	–	–	–	–	–
BQ10	3016	–	*mutL* (aas383Δ1)	–	–	–	*chpA* ^c^ *, fimV* ^c^
BQ11	UK	*mexA* (Q183X)	*mutL* (nt819Δ1)	–	–	–	*pilJ* (nt1122Δ2), *chpA*^c^*, fimV*^c^
BQ12	395	*mexB* (nt2621Δ1)	–	–	–	–	–
BQ13	253	*mexA* (Q46X)	–	–	ΔpslA-pslO (≈278 kb)	*rbdA* (nt190Δ1), *oprF* (Q160X)	*chpA* ^c^ *, fimV* ^c^
BQ14	633	*mexB* (nt41InsGATC)	–	–	–	–	–
BQ15	2431	–	–	–	–	–	–
BQ16	175	–	–	–	–	–	–
BQ17	532	*oprM* (nt1274Δ1)	–	–	–	*rbdA* (Y366X)	*chpA* ^c^
BQ18	554	–	–	–	–	–	*fimV* ^c^
BQ19	253	–	–	–	–	*rbdA* (nt190Δ1), *ladS* (W4X)	*chpA* ^c^ *, fimV* ^c^
BQ20	UK	*mexB* (nt32Δ1)	–	*mucA* (aas56Δ138)	ΔpslA-pslO (≈163 kb)	*rbdA* (aas208Δ1), *oprF* (Q27X)	–
BQ21^b^	480	*oprM* (*nt505*Δ5)	*mutS* (nt1592Δ4)	*mucA* (T96X)	–	–	*fimV* ^c^
BQ22	UK	*mexA* (nt749Δ195)	–	–	–	–	*chpA* ^c^ *, fimV* ^c^
BQ23	357	–	–	–	–	–	*chpA* ^c^
BQ24	2431	*mexA* (nt19Δ1)	–	–	–	–	*chpA* ^c^ *, fimV* ^c^
BQ25	1600	–	–	–	–	–	–
BQ26^b^	UK	*mexB* (Q112X)	–	–	–	–	–
BQ27	379	–	*mutS* (nt339Δ1)	–	–	–	*fimV* ^c^
BQ28	633	*mexB* (nt46InsATCG)	–	–	–	–	–
BQ29	274	*mexA* (nt19Δ2)	–	*mucA* (V147X)	–	–	*chpA* ^c^
BQ30	253	*mexB* (nt283InsG)	–	–	ΔpslA-pslO (≈67 kb)	*rbdA* (nt190Δ1)	*chpA* ^c^ *, fimV* ^c^
BQ31	1637	–	–	–	–	–	*fimV* ^c^
BQ32	253	–	–	*mucB* (L225X)	–	*rbdA* (nt190Δ1)	*chpA* ^c^ *, fimV* ^c^
BQ33	360	*mexB* (nt2169Δ1)	–	–	–	–	*pilJ* (aas672Δ4)
BQ34	1002	*mexB* (Q439X)	–	–	ΔpslA-pslN (≈28.8 kb)		*chpA* ^c^ *, fimV* ^c^
BQ35	3119	–	–	–	–	*rbdA* (nt97InsG)	*chpA* ^c^
BQ36	262	–	–	–	–	–	*chpA* ^c^
BQ37	447	–	–	–	–	–	–
BQ38	UK	–	–	–	–	–	*chpA* ^c^ *, fimV* ^c^
BQ39	585	*oprM* (nt505Δ5)	–	–	–	–	*fimV* ^c^
BQ40	3218	–	–	–	–	*ladS* (nt1345InsAT)	*chpA* ^c^
BQ41^a^	598	*mexA* (Q107X)	–	*mucA* (V147X)	–	–	–
BQ42^a^	395	*oprM* (nt318InsC)	–	*mucA* (V147X)	–	*rbdA* (nt617InsT)	–
BQ43	17	*mexB* (aas739Δ9)	–		–	*ladS* (nt6151Δ1)	*fimV* ^c^
BQ44^a^	UK	–	–	*mucA* (aas191Δ4)	–		*chpA* ^c^ *, fimV* ^c^
BQ45	253	–	–	*mucA* (nt187Δ1)	ΔpslG-pslO (≈9.8 kb)	*rbdA* (nt190Δ1), *ladS* (nt2107Δ1)	*chpA* ^c^ *, fimV* ^c^
BQ46^a^	792	–	–	*mucA* (V129X)	–	–	*fimV* ^c^
BQ47	3511	–	–	–	–	–	–
BQ48	1393	–	–	–	–	–	*chpA* ^c^
BQ49	455	*mexB* (Q106X)	–	–	–	–	*–*
BQ50	253	–	–	–	–	*rbdA* (nt190Δ1), *ladS* (nt1788Δ1)	*chpA* ^c^ *, fimV* ^c^
BQ51	253	–	–	–	–	*rbdA* (nt190Δ1), *ladS* (nt1788Δ1)	*chpA* ^c^ *, fimV* ^c^
BQ52^a^	27	*mexB* (aas766Δ3)	–	*mucA* (V147X)	–	*rbdA* (nt250InsC)	*–*
BQ53^b^	UK	–	*mutS* (nt2550Δ2)	–	–	–	*–*
BQ54	274	–	–	–	–	–	*chpA* ^c^
BQ55	235	–	–	–	–	–	*chpA* ^c^ *, fimV* ^c^

^a^Fenotipo mucoide, ^b^fenotipo SCV, – indica la ausencia de mutaciones, y ^c^ multiples mutacionesde pérdida de función. La abreviatura UK en la columna ST significa ST no conocido.

Entre los 55 aislados secuenciados, el ST más frecuente fue el ST253 (7), seguido de ST395 (3). Siete aislados mostraron una combinación de alelos desconocida, por lo que no se pudieron asignar a ninguno de los secuenciotipos actualmente descritos. Los clones de alto riesgo más frecuentes fueron ST175 (1), ST235 (1), ST244 (1) y ST357 (1), presentando los tres últimos un perfil noMDR. Otros clones de alto riesgo con perfiles no MDR fueron ST17, ST274, y ST532, cada uno de ellos representados por un único aislado.

Las mutaciones de pérdida de función más frecuentes se detectaron en los genes que codifican los tres componentes de la bomba de expulsión MexAB-OprM, detectándose mutaciones en al menos uno de estos genes en 27 aislados.

El hallazgo de aislados con altas tasas de hipermutación es frecuente en infecciones crónicas de *P. aeruginosa *y se debe a la pérdida de función de los genes de reparación del ADN por mecanismo de emparejamieto de bases, *mutS *y *mutL*.En esta cohorte, se identificaron 5 aislados que presentaban mutaciones truncantes en estos genes.

Con respecto al fenotipo mucoide, 17 aislados presentaron mutaciones de pérdida de función en *mucA*, y solo uno en *mucB*. De estos, 10 mostraron un fenotipo mucoide (BQ04-05-06-07-08-41-42-44-46-52). Estos genes codifican proteínas que inhiben la actividad del factor sigma AlgU, que desempeña un papel crucial en la hiperproducción de alginato y la formación de biofilms.

La herramienta IGV se empleó para detectar grandes deleciones en el genoma de *P*. *aeruginosa*. Así, se analizaron grandes edeleciones en los clusteres pel, alg y pel, con funciones todos ellos en la formación de biofilms, así como en los genes relacionados con la síntesis de pioverdina (pvdABCoperon) y su receptor fpvA, ambos implicados en la adquisición de hierro.

En 10 de estos aislados, se detectó la deleción total de dos o más genes del clúster psl, con tamaños que oscilaban entre 1,8 y 278 kb, mientras que los clusteres alg y pel permanecieron intactos en la totalidad de los aislados.

Con respecto a la formación de biofilms, también se analizaron los genes *bifA*, *rbdA*, *oprF *y *ladS *mediante análisis tipo VC.

No se detectaron mutaciones en *bifA*, que codifica una fosfodiesterasa de c-di-GMP que estimula la motilidad en superficie (tipo *swarming*), en detrimento de la formación de biofilms. Por otro lado, RbdA, otra fosfodiesterasa con actividad en la degradación de c-di-GMP, presentó mutaciones de pérdida de función en 12 aislados. Del mismo modo, OprF, la porina predominante de la membrana externa de *P*. *aeruginosa*, presentó mutaciones de pérdida de función en tres aislados. LadS, induce la expresión de genes relacionados con la producción de exopolisacáridos asociados a la formación de biofilms, incluidos Pel y alginato, así como una amplia variedad de factores de virulencia, como los genes con efectos en la motilidad y la expresión del sistema de secreción de tipo III (T3SS). En este estudio, siete aislados presentaron mutaciones truncantes en *ladS*.

Con respecto a la caracterización de la síntesis de pioverdina y su receptor fpvA, todos los aislados a excepción de BQ02-06-07-12-22-25-30-32-42-43-44-45-48-50-51-52 presentaron uno o más genes delecionados del clúster pvd y/o gen *fpvA *(información no incluida en la Tabla 2).

Otras mutaciones de pérdida de función en genes como *pilJ*, *chpA *y *fimV *están relacionadas con la motilidad tipo *twitching*, y podrían ser una adaptación al ambiente viscoso del esputo. En el presente estudio, dos aislados presentaron mutaciones truncantes en *pilJ*, 24 en *chpA*, y 23 en *fimV*. En la Tabla 2 no se muestran las mutaciones concretas en *chpA *y *fimV*, pues son múltiples y de tipo pérdida de función. 

## Discusión

Los resultados obtenidos indican que, a diferencia de otras infecciones respiratorias crónicas, en este conjunto de aislados de *P*. *aeruginosa* causantes de BQ, las tasas de resistencia antibiótica no son particularmente altas. Este escenario brinda la oportunidad única de explorar otros mecanismos de adaptación genética, diferentes de los de resistencia atibiótica, que facilitenn la persistencia de este patógeno en la IBC.

La distribución de los ST refleja la variabilidad genética en la población global de *P.* *aeruginosa.* La identificación de seis aislados no atribuibles a un ST conocido indica que esta población se encuentra en constante evolución genética. Así mismo, la detección de clones de alto riesgo, como ST175, ST235, ST244, y ST357, la mayoría vinculados a perfiles no MDR, evidencia la intrincada dinámica de la diseminación de estos clones y sus posibles efectos sobre el tratamiento de la infección y las estrategias terapéuticas [7].

Con respecto a los mecanismos de adaptación de *P*. *aeruginosa* al ambiente crónico pulmonar, el análisis del genoma de los 55 aislados seleccionados reveló una serie de adaptaciones genéticas significativas que facilitan la persistencia de este patógeno en pacientes con BQ.

El mayor número de mutaciones de pérdida de función se observó en la bomba de expulsión MexAB-OprM, que desempeña un papel fundametal en la resistencia basal a la mayoría de los β-lactámicos, incluidas las nuevas combinaciones de inhibidores de beta-lactámicos/beta-lactamasas. Este hallazgo explica el perfil de hipersensibilidad de estos aislados a estos antibióticos. Además, MexAB-OprM se ha asociado con la virulencia, ya que mutantes deficientes en esta bomba muestran una menor capacidad invasiva. Esto sugiere que las mutaciones truncantes observadas podrían deberse a mecanismos distintos a los relacionados con la resistencia a antibióticos [8].

Con respecto a la formación de biofilms la deleción de genes en el clúster psl, unida a la integridad de los clusteres alg y pel, indica que, en este conjunto de aislados, la formación de biofilms se basa principalmente en los polisacáridos Pel y alginato, en lugar de en Psl [9].

Así mismo, la elevada prevalencia de mutaciones truncantes en el gen *mucA *subraya la importancia de la hiperproducción de alginato en el desarrollo del fenotipo mucoide, un rasgo que determina la virulencia y resistencia de *P. aeruginosa* en las infecciones crónicas [10]. También se ha demostrado que las alteraciones en RbdA y OprF provocan una elevación de los niveles c-di-GMP, lo que deriva en una mayor formación de biofilm a través de la hiperproducción de Pel [9], 11].

Las mutaciones en LadS mejoran la motilidad y actividad de T3SS, en detrimento de la producción de biofilm de Pel y alginato [12]. Cabe mencionar que uno de los cuatro aislados portadores de una mutación de pérdida de función en este gen también presentaba deleción completa de los genes *pslB *y *pslC*, lo que indica que la formación de biofilm podría estar notablemente afectada en este aislado.

Por otro lado, la frecuente deleción de genes asociados a la síntesis de pioverdina y de su receptor FpvA indica que *P*. *aeruginosa* se adapta al huésped favoreciendo rutas alternativas de adquisición de hierro, como podría ser a través del grupo hemo de las proteínas del huésped [13].

Finalmente, se observó un elevado número de mutaciones de pérdida de función en genes asociados a la motilidad tipo *twitching* como *pilJ*, *chpA *y *fimV*. Estas mutaciones se asocian con una motilidad modificada, un fenotipo que se observa también en la FQ, y podría tratarse de una adaptación a la viscosidad del ambiente del esputo [3], 13].

Estos hallazgos genómicos resultan de gran relevancia para mejorar las estrategias terapéuticas en elmanejo de las bronquiectasias.

La deleción de la bomba de expulsión mexAB aumenta la sensibilidad a los β-lactámicos, por lo que si se conoce este hallazgo, pueden diseñarse terapias antimicrobianas más específicas[8]; asimismo la reducción de la biosíntesis de pioverdina indica un cambio en la captación de hierro,evidenciando así el potencial de las terapias de quelación de hierro para interferir en su metabolismo y en la formación de biopelículas.

Además, dada la rapidez con la que *P. aeruginosa *ha desarollado resistencia a los antibióticos, limitando así la eficacia de estos a largo plazo, se están investigando tratamientos no antibióticos, como la terapia con fagos [14], los inhibidores de *quorum sensing* y las terapias basadas en nanopartículas [15], como tratamientos alternativos a las terapias tradicionales o bien como adyuvantes.

En conclusión, la caracterización genómica de *P*. *aeruginosa* resulta esencial para identificar los mecanismos que permiten su adaptación al ambiente pulmonar crónico y facilitar el desarrollo de terapias alternativas que minimicen la estrategia de “prueba y error” que se sigue aplicando actualmente.
